# Experiences of general practitioners, home care nurses, physiotherapists and seniors involved in a multidisciplinary home-based fall prevention programme: a mixed method study

**DOI:** 10.1186/s12913-016-1719-5

**Published:** 2016-09-05

**Authors:** Astrid E. Amacher, Irina Nast, Barbara Zindel, Lukas Schmid, Valérie Krafft, Karin Niedermann

**Affiliations:** 1 Zurich University of Applied Sciences, Institute of Physiotherapy, Technikumstr. 71, Winterthur, 8401 Switzerland; 2 Swiss League Against Rheumatism, Josefstr. 92, Zurich, 8005 Switzerland; 3 Department of Rheumatology, Central Hospital of Lucerne, 6000 Lucerne 16, Switzerland

**Keywords:** Fall prevention, Elderly, Multidisciplinary care, Physiotherapy, Implementation

## Abstract

**Background:**

The feasibility of effective fall prevention programmes (FPPs) for use in daily clinical practice needs to be assessed in the specific healthcare settings. The aim of this study was to explore the perceived benefits and barriers of an evidence-based, home-based pilot FPP among the involved seniors, general practitioners (GPs), home care nurses (HCNs) and physiotherapists (PTs), in order to develop tailored implementation strategies.

**Methods:**

The study was a mixed method study using an ‘exploratory sequential design’. In the initial qualitative sequence, semi-structured interviews were performed with four participants from each group and analysed using a deductive content analysis. In the successive quantitative sequence, target group specific postal surveys were conducted with all participants. The triangulation of both steps allowed merging the in-depth experiences from the interviews with the general findings from the survey.

**Results:**

In this evaluation study participated 17 seniors (mean age 79.7 (SD +/-6.2) years). 40 GPs, 12 HCNs and four PTs. All were satisfied with the organization and processes of the FPP. The main benefit, perceived by each target group, was the usefulness of the FPP in detecting risk of falling at the senior’s home. A low number of recruiting GPs and HCNs, divergent opinions of the health professionals towards the aim of the FPP as well as no perceived need for changes by the seniors were the most important barriers to the participation of (more) seniors.

**Conclusions:**

Multidisciplinary home-based fall prevention is a useful approach to detect the risk of falling in seniors. The barriers identified need to be resolved through tailored strategies to facilitate the successful nationwide implementation of this pilot FPP.

**Electronic supplementary material:**

The online version of this article (doi:10.1186/s12913-016-1719-5) contains supplementary material, which is available to authorized users.

## Background

Falls by the elderly frequently result in injury and represent one of the most common and serious public health problems in Switzerland [1]. Around 30 % of community-dwelling persons over 65 years fall each year. This incidence rate rises by 10 % with each decade of increasing age [2]. The risk of recurrent falls is 50 % [3]. In 2013, 38.5 % of the people over 65 years were aged 80 years or older [4]. This demographic development, in combination with the age-related rise in the fall incidence rate, results not only in greater health problems and an increased requirement for care and fall prevention programmes (FPPs) but also in higher socio-economic costs [5, 6].

Research consistently showed encouraging results for multifactorial and multidisciplinary FPPs [6–11]. Other studies concluded, through cost-benefit analysis of community-based FPPs targeted at older people at all risk levels, that well-designed programmes for the elderly were highly cost effective [7, 12]. However, a trial by Hendriks et al. [13] showed substantial discrepancy between the FPP under study conditions and the same FPP implemented in daily practice. The authors recommended the assessment of the feasibility of such programmes for clinical practice and underlined the importance of implementation research in the specific healthcare setting, with special attention to barriers, e.g. the reasons for insufficient adherence of participants to fall prevention recommendations.

The Swiss League Against Rheumatism (SLAR) therefore conducted a multifactorial and multidisciplinary pilot FPP in Central Switzerland. It was based on the Australian ‘Stay on Your Feet SOYF’ FPP (1992–1996), where general practitioners screened their seniors > 60 years for fall risk and enrolled them to the SOYF. The SOYF addressed footwear, vision, physical activity, balance and gait, medication use, chronic conditions, plus home and public environmental hazards. This programme was evaluated extensively and achieved a significant reduction in fall-related hospital admissions [11, 12, 14]. The Swiss pilot FPP addressed older seniors living independently with or without previous falls. It was supported by a large body of stakeholders in this region: the association of general practitioners (GPs); the organization of home care nurses (HCNs), i.e. nurses and home helpers; the central hospital; the age organization “Pro Senectute”; the platform ‘Osteoporosis’ of the Swiss Society for Rheumatology and the section “health in age” of the public health department.

All HCNs (particularly the home helpers) were asked to assess the risk of falling among their clients (older seniors living at home, at risk of falling or with previous falls). In the case of a positive risk assessment, they were required to send a notification to the senior’s GP and the SLAR. In case the GP included a senior in the FPP (either referred by the HCN or assessed by him/her), the GP was also to inform the SLAR. The SLAR then made contact with one of four physiotherapists (PTs) who were specially trained for this FPP. The PT visited the senior at home and performed a detailed assessment of her/his risk of falling, eliminated the identified environmental risk factors and provided tailored exercises [15]. The PTs informed the GPs about the assessment results and the measures taken and provided recommendations for further action in a report.

The SLAR as a national organisation will take care of the nationwide implementation of this FPP after its evaluation. Implementation is the planned and systematic approach with clear strategies for dissemination, implementation and/or maintenance of innovations or changes in (clinical) practice and encompasses a step-by-step procedure: After defining the targets for improvement or change, an ‘analysis of current (clinical) performance, target group and setting is performed, including the exploration of facilitators and barriers for change among the target groups or stakeholders, followed by ‘the development of tailored strategies and measures to change practice’, subsequent ‘execution of this implementation plan and finally its ‘evaluation and adaptions if necessary’ [16]. Related to this pilot FPP, the analysis of the current practice showed that there was no such home-based FPP available, and that thus this pilot FPP was innovative. The next step, which was the focus of this research, was to analyse the facilitators and barriers among the target groups of this FPP. Facilitators and barriers are generic, i.e. they may occur in any implementation process, or specific to the specific innovation being implemented. They are related to the context, i.e. to a) the individuals (health professionals); b) social setting (seniors, professional colleagues), c) organisational factors (management) or d) economic and system factors such as money or laws [17]. Facilitators may provide promising approaches and act as ‘selling points’, whilst barriers anticipate challenges and require tailored strategies.

Therefore this evaluation study was conducted simultaneously to the pilot FPP with the aim of investigating the experiences of the seniors, GPs, HCNs and PTs and identifying and analysing the facilitators and barriers of the FPP. As main facilitators we a priori assumed “satisfaction with the project” and “benefits of the project”; as main barriers we assumed reasons related to the inclusion and participation in the FPP. The results of this analysis will be linked to the factors a) to d), in order to develop tailored implementation strategies.

## Methods

### Study design

This study is a mixed method study with an ‘exploratory sequential design’ according to Creswell and Plano Clark [18]. An initial qualitative sequence was followed by a quantitative sequence. The qualitative phenomenological sequence (sequence 1) used semi-structured interviews to obtain greater and more differentiated information than would have been possible through a questionnaire. The subsequent quantitative sequence (sequence 2) was based on these interview findings and utilized group-specific questionnaires in order to validate the qualitative results. The triangulation of both steps, i.e. the merging of the in-depth opinions obtained from the interviews with the larger scale findings from the survey, strengthens the validity of the results.

### Participants

The four target groups consisted of the GPs, the HCNs, the PTs and the seniors involved in the FPP. In sequence 1, four persons from each target groups (total *n* = 16) were selected for interviews, to achieve a broad range of demographic characteristics, in terms of gender (in seniors and general practitioners (there was no choice in HCNs and PTs) and region (urban or rural in all target groups). In sequence 2, all GPs and HCNs in the region, as well as seniors, after providing written informed consent to participation, received group-specific questionnaires. The four physiotherapists were all interviewed and therefore not involved in step 2.

### Procedure

#### Qualitative phase

The questions for the semi-structured interviews were developed based on literature [19] and expert opinions and tailored to each target group. They encompassed four a priori defined points of interest, representing both facilitators and barriers for future implementation of the FPP: 1. satisfaction with the organization and processes of the programme; 2.strength and benefits of the programme; 3. barriers to the inclusion of seniors and 4. barriers to the participation of seniors. The interview questions were pretested with one member of each target group.

The four selected GPs, HCNs and PTs were interviewed by telephone, the seniors face to face. The interviews were conducted in Swiss German, audiotaped and lasted 25 min on average. Transcription was conducted verbatim by using a predefined list of criteria adapted from Dresing and Pehl [20]. The language was changed from Swiss German to German after analysis of the data and from German to English during the writing of the manuscript.

#### Quantitative phase

The target group specific questionnaires were developed by two experts based on the deductive content analysis (see next paragraph) of the interviews [21]. They consisted of 8 categories/63 and 8 categories/61 questions for the GPs and the HCNs respectively; and 6 categories/40 questions for the seniors, all with dichotomous answer options (0 = no, 1 = yes).

The questionnaires were sent by post to the GPs, HCNs and seniors with the request to return the completed questionnaires within four weeks, using the enclosed postage-paid envelope. The chair persons of the GP association and the HCN organization reminded their members by e-mail after one and three weeks. Seniors were personally reminded by their PT.

In addition to the qualitative and quantitative data obtained in the two phases, characteristics of the participating persons were obtained from the SLAR.

### Data analysis

A deductive content analysis was performed on the interview data [21, 22]. The transcripts were allocated line-by-line in a deductive manner to the corresponding a priori defined points of interest, resulting in ‘meaning units’. These were condensed into ‘condensed meaning units’ and finally into ‘subcategories’ [23] (see Additional file 1). The target group specific questions for the questionnaire were developed from identified subcategories.

Descriptive statistics were performed using SPSS software, version 21 (SPSS, Chicago, IL).

### Triangulation

The presentation of the following results is structured by five points of interest: Four of them were defined a priori, the fifth emerged from the data: 1. satisfaction with organization and processes of the FPP; 2. strength and benefits of the FPP; 3. barriers to the inclusion of seniors; 4. barriers to the participation of seniors; and 5. barriers in interdisciplinary cooperation. In each section, the interview results (sequence 1) are explicated and emphasized by the participants’ statements. Additionally, they are validated, i.e. supported or not, by the survey results (sequence 2), thus performing the triangulation of both steps. Tables 3, 4, 5, 6, and 7 depict a selection of questions per point of interest and subcategory, derived from the condensed meaning units in the qualitative data, along with the quantitative survey’s results. All questions derived from qualitative data and results of the quantitative survey are provided in the Additional files.

## Results

### Participants

**Fig. 1:**
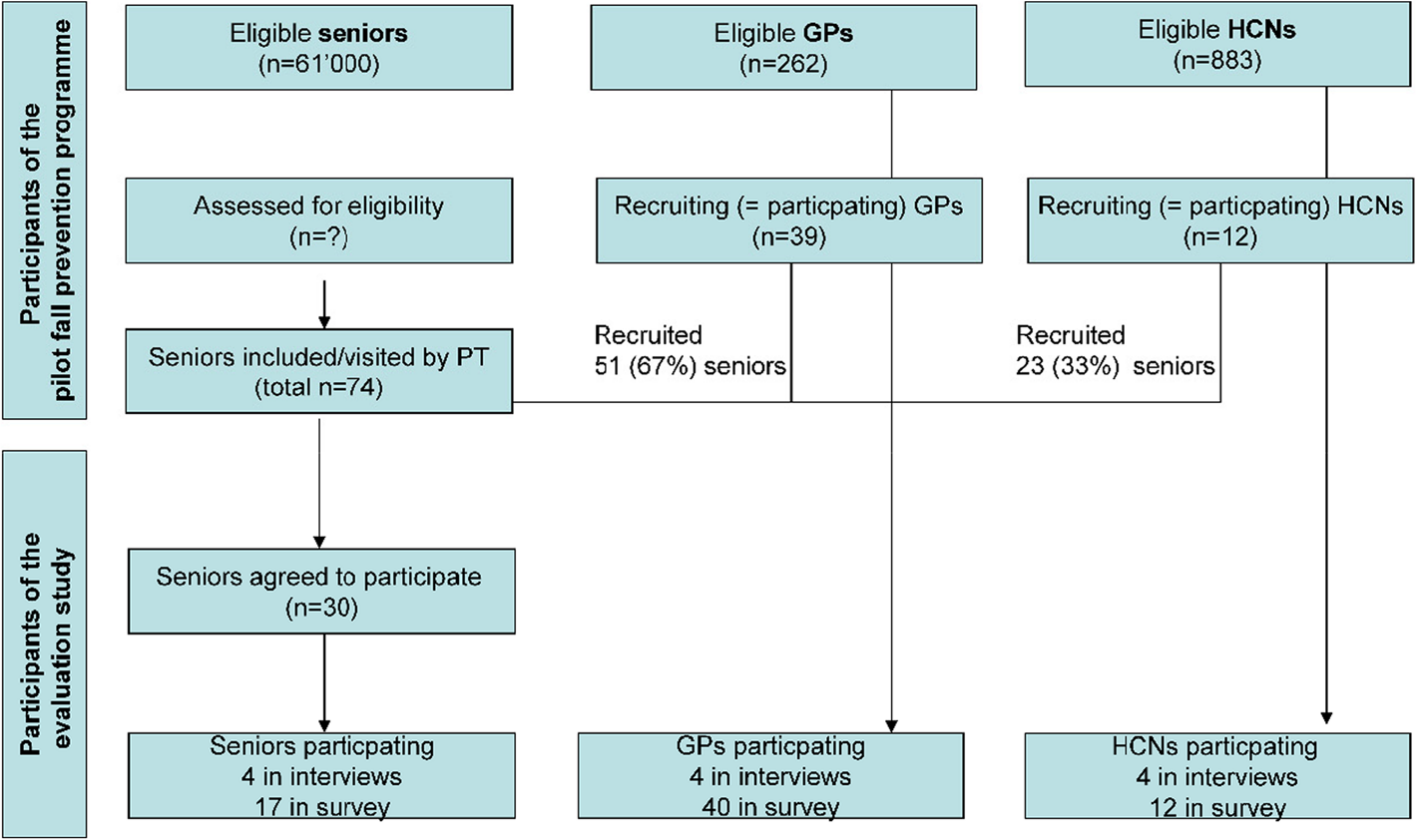
Flow chart of pilot FPP and evaluation study

The flow chart in Fig. 1 provides an overview on the FFP-study populations and the number of participants included in qualitative interviews and in the quantitative questionnaire survey.

From more than 61’000 potential fallers over 65 years of age in this region [24], 74 seniors were recruited by GPs and HCNs to the FPP over one year; 51 (69 %) of them by 39 GPs (28 % of the 262 GPs in the area) and 23 by 12 HCNs (1.5 % of approximately 826 HCNs). Interviews were conducted with four participants of each target group. Participants’ characteristics are shown in Table 1.

**Table 1:** Demographics of interview participants

	Seniors	PTs	GPs	HCNs
Age (in years): mean (range)	85 (65–88)	55 (49–59)	54 (49–60)	50 (48–54)
Gender (F/M)	2/2	4/0	1/3	4/0
Area (urban/rural)	2/2	NA	3/1	1/3
Practice (in years): mean (range)	NA	32 (29–40)	24 (14–36)	10 (3.5–20)

*GP* general practitioner, *HCN* home care nurse, *PT* physiotherapist, *NA* not applicable

Of the 74 seniors recruited, 32 (53 %) agreed to answer the questionnaire survey, 17 (mean age 79.7 (SD+/−6.2) years) finally returned the questionnaire. Of all 262 GPs, 40 (15 %) participated in the survey; 25 of them stated being familiar with the FPP and could answer all questions. The 15 GPs who were not familiar with the FPP only answered the first three general questions: 1) familiarity with present fall prevention programme; 2) relevance of fall prevention in seniors over 65; 3) public attention of fall prevention. All 12 HCNs who had recruited seniors to the FPP returned the questionnaires. The characteristics of the questionnaire survey participants are shown in Table 2.

**Table 2:** Demographics of survey participants

	Seniors (*n* = 17)	GPs (*n* = 25)	HCNs (*n* = 12)
Age (in years): mean (SD)	79.7 (6.2)	44.2 (9.1)	53.9 (9.8)
Gender n (%)			
Female	13 (76.5 %)	7 (17.5 %)	11 (91.7 %)
Male	4 (23.5 %)	33 (82.5 %)	1 ( 8.3 %)
Area			
Urban	4 (23.5 %)	12 (30 %)	4 (16 %)
Rural	13 (76.5 %)	28 (70 %)	8 (32 %)
Practice (in years): mean (SD)	NA	10.8 (8.2)	18.6 (11.1)

*GP* general practitioner, *HCN* home care nurse, *PT* physiotherapist, *NA* not available/applicable, *SD* standard deviation

### Satisfaction with organization and processes of the FPP

For this a priori defined point of interest, no subcategories were revealed from the interview data. The HCNs, GPs and PTs underlined the good organisation of the project with adequate information, helpful documents and a well acceptable expenditure of time required for the project participation: *“I liked the good information: It was functional, and we received these lovely flyers (…). I considered this material to be easy to fill in and to register the seniors.”* (HCN4). The seniors in the interviews however, although they did not express a lack of information, did not seem to have been informed sufficiently about the process: *“Eh, what kind of information? (…) She* (the physiotherapist) *just said that she would like to include me into her project and that she would like to do some assessments, to test my skills.”* (senior 3). Similarly, the GPs did not reveal a need for more information, but they seemed not very well informed about the project either. One of their respondents explained how difficult it is for the GPs to overview all projects running: *“We are inundated* (with prevention projects) *and sometimes, I think all the activities are somehow excessive.”* (GP 2). Survey results (Table 3) supported the findings of the qualitative data on the satisfaction of the HCNs and the GPs; however, the majority of seniors and GPs did not express a lack of information in the survey.

**Table 3:** Selection of detailed questions on the topic “Satisfaction with the organization and processes of the FFP” and survey results

	Ratings from the survey		
	Seniors (*n* = 17)	GPs (*n* = 25)	HCNs (*n* = 12)
	“yes” *n* (%)	“yes” *n* (%)	“yes” *n* (%)
Were you satisfied with the organization of the project?	15 (88 %)	16 (64 %)	12 (100 %)
Were you well informed before the start of the project?	15 (88 %)	16 (64 %)	12 (100 %)
Was the expenditure of time for project participation adequate?	15 (88 %)	25 (100 %)	9 (75 %)

### Strength and benefits of the FPP

Qualitative data revealed the following subcategories of “strengths and benefits of the FPP”: “General and specific benefits perceived by seniors”, “Interests of seniors”, “Further offers desired by seniors”, “PTs instructions followed by the seniors” and “Project benefits perceived by GPs and HCNs”.

One central benefit of the FPP in the eyes of the seniors was the recognition of their own risk of falling. Some respondents were able to follow the exercise instructions given by the PT on the basis of the risk assessment, and they experienced improvements: *“This* (performing the exercises) *has already helped nicely (…) I am now able again to rise from the floor without assistance.”* (senior 2). But, some seniors didn’t feel capable to perform the instructed exercises (*“I had a serious conversation with my doctor: My body tells me a story contradictory to the good advice I received* (by the physiotherapist)” (senior 1). And not all respondents consequently followed the advice to minimize environmental risk factors at home: *“I already said at the beginning that she* (the physiotherapist) *may come* (to my home)*, but that I will not remove any carpets”* (senior 4). Veritable interests of seniors related to their participation were the recommendation of the GP or HCN, the recognition of risk for falling and their interest in the degree of the personal risk of falling. However, for most of the respondents, the FFP provided enough benefit; they did not require further visits or regular support. The programme benefits perceived by GPs and HCNs met the primary goals of the FPP, as the programme in their eyes was useful to prevent falls, draw attention to the risk of falling and to detect sources of risk of falling. Survey results (Table 4) supported these findings from qualitative data.

**Table 4:** Subcategories (bold) of and a selection of detailed questions on the topic “strengths and benefits of the FFP” with survey results

	Ratings from the survey		
	Seniors (*n* = 17)	GPs (*n* = 25)	HCNs (*n* = 12)
	“yes” *n* (%)	“yes” *n* (%)	“yes” *n* (%)
**General and specific benefits perceived by seniors**			
Was the personal visit of the PT at your home helpful?	13 (76 %)	NA	NA
Did you recognize your own risk of falling due to the consultation by the PT?	14 (82 %)	NA	NA
**Interests of seniors**			
Why did you participate?			
- *GP or HCN recommended it to you.*	10 (59 %)	NA	NA
- *You recognized the risk of falling and have been motivated to do something against it actively*.	8 (47 %)	NA	NA
- *You have been interested in the degree of your own risk of falling*.	8 (47 %)	NA	NA
- *Because of your confidence in the HCN.*	6 (35 %)	NA	NA
**Further offers desired by seniors**			
Would you participate again if you had the possibility to do so?	9 (53 %)	NA	NA
**PTs instructions followed by seniors**			
Did you investigate changes in your home after the consultation by the PT (i.e. fixating carpets or signalize door sills)?	10 (59 %)	NA	NA
Do you execute the instructed physical exercises received from the PT?	10 (59 %)	NA	NA
Do you carry out further measures such as group therapies or physiotherapy after the consultation by the PT?	6 (35 %)	NA	NA
**Project benefits perceived by GPs and HCNs**			
Was the project useful to:			
- *Prevent falls?*	NA	20 (80 %)	10 (83 %)
- *Draw attention to the risk of falling?*	NA	21 (84 %)	10 (83 %)
- *Detect sources of risk of falling?*	NA	18 (72 %)	8 (67 %)

### Barriers to the inclusion of seniors

The interviews with GPs, HCNs and seniors revealed three subcategories of “barriers to the inclusion of seniors”: 1) “Lack of clarity regarding the aim of the programme”, 2) “Procedural approach of GPs and HCNs” and 3) “Reasons of GPs for not recruiting seniors”. The GPs mostly stated that the FPP should primarily prevent *first* falls, whilst the HCNs rather thought that the focus ought to be prevention of *further* falls. The sub category, “procedural approach of GPs and HCNs", was presumably linked to this lack of clarity: The majority of them knew how to recruit, but they rather seldom did it. They recruited their clients on the basis of different idiosyncratic selection criteria, such as the seniors’ known falls, their obvious risk of falling or their mobility problems: *“The criterion* (to recruit seniors) *is my own observation.”* (GP2). *“It is obvious at the patients’ gait. How, when I get her in the waiting room, how she is walking or sitting down. That she’s obviously a candidate for falling.”* (GP 3).

Some of the GPs prompted their patients to register themselves for the project. It has to be supposed, that this requirement to self-register was a barrier for some of these patients: “*It was disappointing to discover that several seniors to whom I had distributed the registration forms did not register…. I wanted them to do it by themselves at home.” (GP 4).* For GPs, the main reason not to recruit more seniors was not, as it could be expected, the expenditure of time, but rather the anticipated reaction of “no need” or “refusal” by seniors. On the other hand, GPs and HCNs seemed to have a great influence on the seniors’ decision to register for the project: *“And she said this project is supported by the HCNs community. So I said, then I will participate.”* (senior 1). Therefore, if GPs and HCNs did not recognize the need for participation (e.g. for the reason, that they assume another target group), this was an important barrier. Other reasons for the restraint recruitment of GPs were that the project operations were not clear or that registration forms were not available. Survey results (Table 5) supported these findings.

**Table 5:** Subcategories (bold) of and a selection of detailed questions on the topic “Barriers to the inclusion of seniors” with survey results

	Ratings from the survey		
	Seniors (*n* = 17)	GPs (*n* = 25)	HCNs (*n* = 12)
	“yes” *n* (%)	“yes” *n* (%)	“yes” *n* (%)
**Lack of clarity regarding the aim of the project**			
What is the primary aim of the project (one answer):			
- *The prevention of first falls.*	NA	20 (80 %)	4 (33 %)
- *The prevention of further falls.*		6 (24 %)	8 (67 %)
**Procedural approach of GPs and HCNs**			
Did you know how to recruit seniors?	NA	21 (84 %)	12 (100 %)
Did you use reminders (i.e. flyer, post-it…)?	NA	4 (16 %)	4 (33 %)
**Reasons of GPs for not recruiting seniors**			
**Why did you not recruit any seniors? (** ***n*** **= 13)**			
- *No perceived need/refusal by senior*.	NA	10 (77 %)	NA

### Barriers to participation of seniors

Within the point of interest “Barriers to participation of seniors”, two subcategories derived from the interviews: “Personal reasons of seniors” and “Barriers for PTs to do assessments and give instructions.” Physiotherapists, GPs and HCNs speculated in the interviews on personal reasons for seniors not to participate in the FPP. However, reasons such as “having difficulties with being consulted at home” or “feeling urged to participate” were hardly ever mentioned by the seniors as reasons for non-participation. Project costs on the other hand would be a barrier at least for some seniors. This concern was raised in the interviews by the health professionals, and it was confirmed by the seniors in the survey (Table 6): only one third of the seniors rated that they would have participated, even if they had to pay for it. “Barriers for PTs to do assessments and give instructions” accrued from the circumstance that some seniors were either not capable anymore to do the assessments and engage in exercises (this finding has also been supported by the survey results) or that their home had been checked before for sources of risk of falling by the HCN. *PT 3: “I had the feeling that if an HCN recommended a senior for the FPP, I could hardly do any preventive intervention, because much of it was already covered. If a GP recruited the senior it was different, because I could still do a lot.”* The HCNs indeed stated that they always performed fall prevention at a senior’s home, i.e. elimination of environmental risk factors, irrespective of this FPP.

**Table 6:** Subcategories (bold) of and a selection of detailed questions on the topic “Barriers to participation” with survey results

	Ratings from the survey		
	Seniors (*n* = 17)	GPs (*n* = 25)	HCNs (*n* = 12)
	“yes” *n* (%)	“yes” *n* (%)	“yes” *n* (%)
**Personal barriers for seniors**			
Had you participated in the project in case you had to pay for it?	6 (35 %)	NA	NA
**Barriers for PTs to do asssessments and give instructions**			
Was it possible to perform physical assessments to obtain your risk of falling?	10 (59 %)	NA	NA

### Barriers in interdisciplinary cooperation

This category was added after a first round of analysis of the qualitative data, as barriers in interdisciplinary cooperation emerged on several areas: “Satisfaction with multidisciplinarity”, “Consideration of other professionals’ opinions”, and “Information and processes”. Although in the interviews, HCNs uttered only initial doubts about the multidisciplinary FPP, and although GPs and HCNs expressed in their majority satisfaction with their role allocated in the project, only a minority of them reported to be satisfied with the multidisciplinary setting in the survey. Reservations regarding multidisciplinarity may have risen from the fact that areas of competences were overlapping in this field: *“Consulting in general is very important to us HCNs. We always perform a medical diagnostic screening and look also for these things. (…) ... and consulting* (regarding facility)*.* (We say)*: “You have this carpet”, then we solve this* (problem) *or search for solutions. Also the risk of falling in the shower.”* (HCN 1). Furthermore, GPs and HCNs were not always satisfied with the reports they received from the physiotherapists (unfortunately, they did not mention this fact in the interviews), and they often did not implement PTs recommendations. Finally, the information of other stakeholder groups was sceptically evaluated: *“It was put about that GPs are informed, but however, our GPs did not really have a clue. (…) But the project flyer (previously mentioned) was helpful then.”* (HCN 1). Altogether, the statements on the multidisciplinary cooperation were slightly more positive in the interviews than they were subsequently rated in the survey (Table 7).

**Table 7:** Subcategories (bold) of and a selection of detailed questions on the topic “Barriers in interdisciplinary cooperation” with survey results

	Ratings from the survey		
	Seniors (*n* = 17)	GPs (*n* = 25)	HCNs (*n* = 12)
	“yes” *n* (%)	“yes” *n* (%)	“yes” *n* (%)
**Satisfaction with multidisciplinarity**			
Was it positive that the project was multidisciplinary?	NA	9 (36 %)	4 (33 %)
Were you satisfied with the role allocation in the project?	NA	15 (60 %)	8 (67 %)
Would you support the participation of e.g. rehabilitation centers or hospitals in the project?	NA	9 (36 %)	9 (75 %)
**Consideration of other professions’ opinions**			
Were you satisfied with the report received from PTs?	NA	9 (36 %)	6 (50 %)
Did you partially or in general implement the recommendations by the PTs?	NA	11 (44 %)	7 (58 %)
**Information and processes**			
Do you think GPs were well informed?	NA	NA	6 (50 %)
Were your medical practice assistants informed?	NA	8 (32 %)	NA

The Additional files complete the information of the Tables S3-S7 and encompass: the interviews' content analysis (Additional file 1); all questions on the topics with survey results (Additional file 2); the SPSS databases (in excel format) of the survey results from the health professionals (Additional file 3) and seniors (Additional file 4).

### Allocation of facilitators and barriers to context factors (individual/social/organizational/system)

Facilitators (“Satisfaction with organization and processes” and “Strength and benefits of the FPP") and barriers (“to the inclusion of seniors”, “to participation of seniors” and “in interdisciplinary cooperation”) were allocated to the context factors. They require different strategies to implement the FPP (displayed in Table 8).

**Table 8:** Allocation of identified facilitators and barriers to the context factors (individual/social/organizational/system) and suggestions for implementation strategies

Context/Point of interest	Individual	Social	Organizational	System
Satisfaction with organization and processes of the FFP	F: Satisfaction of HCNs and seniors with information and organization F: Satisfaction of GP with expenditure of time for project participation F: High satisfaction of physiotherapists with time allocated for the home visit *→ Highlight satisfaction of physiotherapists, HCNs and seniors*		B: Seniors and GPs not sufficiently informed *→ Information strategy tailored to target group*	F: FFP is funded B: Concerns for the future *→ Programme funding has to be granted for the future*
Strength and benefits of the FFP	F: Majority of seniors perceives general and specific benefits (such as insight into risk of falling); a concise majority executes instructed exercise and changes in their homes *→ Strengthen motivation and self-efficacy in seniors*	F: Potential of the FPP to prevent falls and draw attention to the risk of falling accepted by GP and HCNs *→ Highlight confidence of GPs and HCNs in potential effectiveness of the FFP*		
Barriers to the inclusion of seniors	B: Seniors don’t need or/and refuse participation *→ Highlight the importance that GP and HCNs invest efforts at convincibility, as they exert a great influence in their patients/clients.* *→ Invest in information, awareness rising; self-efficacy, empowerment of seniors*		B: Lack of clarity regarding the aim and target group of the project; B: no systematic recruitment procedure *→ Invest in clear messages about the aim of the project and in clear recruitment instructions*	
Barriers to participation			B: Recruited seniors are not capable to do exercise or their home has already been checked for extrinsic risk factors *→ Invest in clear messages about the aim of the project and in clear recruitment instructions*	B: Taboo character B: Costs: Participation not for free *→ Programme funding has to be granted for the future*
Barriers in interdisciplinary cooperation		B: Unsatisfactory information-flow *→ Invest in clear information, who should be informed when and how about the project*	B: Procedure reports *→ Invest in clear instructions how to proceed with reports*	

*F* facilitator, *B* barrier

## Discussion

This study aimed to explore the individual, structural and process-related facilitators and barriers of a pilot FPP in Switzerland in order to support its nationwide implementation. The majority of all involved persons, the health care providers as well as the seniors, were satisfied with this project aiming at preventing falls through the detection and elimination of risks at the seniors’ homes. These confirmed facilitators will certainly work as strong pros and selling arguments in the planned implementation process.

A low number of recruiting GPs and HCNs, divergent opinions of the health professionals towards the aim of the FPP, as well as no perceived need for changes by the seniors were the most important barriers to include (more) seniors.

The allocation of facilitators and barriers to the individual, social, organizational and system context factors provides the basis for developing tailored strategies when executing the implementation plan.

One important indicator for success of such a FPP is the number of seniors who participate, which may also be determined by the number of recruiters who actively include seniors. The data showed that only 74 of more than 61’000 potential fallers in the area were recruited to the FPP over one year, by a minority of only 15 % and 1.5 % of the participating GPs and HCNs respectively. This pilot FPP was supported by a large body of stakeholders in the region and extensive written and oral information about the project was provided to the GPs and HCNs before the start which we judge as strong facilitators. However, not all GPs and HCNs were familiar with the FPP. This emphasises the strategy to carefully tailor the information to each target group.

The perceived strengths and benefits of the pilot FPP were limited by the divergent opinions as to which seniors would benefit the most from the FPP. The majority of the HCNs stated that the FPP should prevent further falls in recurrent fallers, whilst the PTs and the majority of the GPs indicated to prioritize first fall prevention. However, the GPs almost exclusively recruited seniors who were frail and in old age. They explained their strategy with the fact that younger and healthier seniors usually did not perceive a need for joining the FPP. This discrepancy is a specific barrier and a great challenge inherent in FPPs. For the successful implementation of this FPP, this issue needs to be resolved by a clear message of the aims and target groups and clear instructions about the recruitment procedures. Recent findings show that good physical and cognitive functional abilities may be strong predictors of adherence in multifactorial FPPs [25] and that FPPs for persons at high risk of falling are not cost-effective because of an increased need for further therapies, medication, healthcare devices, aids, adaptations and low adherence to the recommendations [26]. Most participants of the FPP reported an increase of awareness towards their risk of falling, but not all participants really wanted (or were able) to follow the instructions and only a part of them reported to be adherent to the advice and exercises after the visit. This leads again to the question if the target group was adequate. It is not an easy task, besides the usual time and routine constraints in daily clinical practice, to involve pre-frail seniors with low risk of falling. However, to overcome this barrier it is important that GP and HCNs become aware of the great influence they have on their patients and go to the time and efforts of convincing them. The central message in the recruitment of younger, pre-frail seniors would be that FPPs (or rather ‘gait security and mobility programmes’) lead to better health and longer independency [19]. For a FPP offering an intervention at the senior’s home, this may be a strong and consistent message. Positive goals (what to reach) are more successful than negative ones (‘what to avoid’) [27] and may also help to overcome the taboo character of ‘falls’ and ‘fall prevention’ in our society and most of all in the seniors themselves.

Nearly a third of the GPs wanted their patients to register for the FPP themselves, which meant an unnecessary additional barrier for the seniors. Instead, the often revealed lack of insight concerning the need of fall prevention among seniors would have required a high degree of support in the registration procedures [19]. This role could be fulfilled by medical practice assistants (MPAs). During the pilot programme, they were involved only in a minority of the GP practices. The information flow between GP and MPA, as well as between GP and HCN turned out to be important barrier. The strategy for implementation could be to invest in clear information, and who should be informed when and how about the project. The MPAs for example may be very important to overcome recruiting problems and the constraints in time of the GPs. Well-informed and trained MPAs may substantially support the GPs and seniors alike and take care of a smooth recruitment process.

Programme costs to be covered by the participants themselves are usually a key barrier. This was not the case in this pilot FPP, as without costs for the participants. However costs may be a future barrier for seniors to participate in this FPP. Only a minority of the responding seniors would participate if they have to pay by themselves. Finding continuous financial resources for this FPP may therefore be of capital importance to its successful implementation. A FPP is not covered by the basic health insurance in Switzerland, although health insurances in fact ought to have an interest in supporting this evidence-based, low-threshold FPP. Therefore, mid-term changes of the payment for preventive care, such as FPPs, are mandatory, given the high socio-economic costs of falls.

Strengths of this study are that all stakeholders were involved in this evaluation and the use of an exploratory sequential design, including triangulation strengthened the validity of the results. However it is a limitation of this study that the barriers to participation in the FPP were only derived from statements of the involved persons. Therefore, information from seniors who declined to participate in the programme as well in this evaluation study is not available. This target group would have been difficult to reach but could have provided valuable information. The same would be true for information gained from GPs or HCNs who did not participate. Interestingly all twelve HCNs, participating in the programme, also answered the survey. However they only represented a small minority of all HCN branches and staff. The lack of HCN-branches which didn’t recruit any seniors could have biased the results. Knowing about the reasons for non-participation of the other 98.5 % may be very relevant for the future of this FPP.

Further research should include investigating other recruitment strategies, involving other health professionals, e.g. MPAs, and evaluating the effect of the PT intervention, including their reports to the GPs, as well as the cost effectiveness of the FPP.

## Conclusion

The allocation of the facilitators and barriers to the individual, social, organizational and system context factors provides the basis for tailoring the strategies when executing the implementation plan. Mainly the barriers require specific strategies: The low number of recruiting GPs and HCNs should be addressed by improving the information flow between the involved health professionals and by thinking about alternative recruitment channels. The divergent opinions of the health professionals towards the aim of the FPP need to be resolved by a clear message of the aims and target groups and clear instructions about the recruitment procedures. A careful but convincing communication, emphasizing long-term gait security and independence, by the health professionals and especially the GPs may be a key to overcome the not perceived need for changes by the seniors.

## Additional files

Additional file 1: Data file interviews’ content analysis. (DOCX 45 kb) Additional file 2:
**Tables S3**-**S7.** All detailed questions on the topics with survey results, in addition to the Tables 3, 4, 5, 6 and 7 included in the main file. (DOCX 26 kb) Additional file 3: SPSS data file health professionals. (XLS 71 kb) Additional file 4: SPSS data file seniors. (XLS 19 kb)

## Abbreviations

FPP(s): Fall prevention programme(s)
GP(s): General practitioner(s)
HCN(s): Home care nurse(s)
MPA(s): Medical practice assistant(s)
PT(s): Physiotherapist(s)
SD: Standard deviation
SLAR: Swiss League against rheumatism
SOYF: Stay on your feet
