# Supplementary material for: Experiences of general practitioners, home care nurses, physiotherapists and seniors involved in a multidisciplinary home-based fall prevention programme: a mixed method study
Source: BMC Health Serv Res. 2016 Sep 5;16(1):469. doi: 10.1186/s12913-016-1719-5 (PMC5011799; doi:10.1186/s12913-016-1719-5)
Supplement: Additional file 1: — Data file interviews’ content analysis. (DOCX 45 kb) [file 12913_2016_1719_MOESM1_ESM.docx]

| Results Qualitative Sequence (interviews): Content Analysis |
| --- |

| 1. Satisfaction with organization and processes of the fall prevention project (FPP) | | |
| --- | --- | --- |
| *Meaning unit* | *Condensed Meaning Unit* | *Preliminary Questions derived from meaning units* |
| - Organisation was very good | Organisation | - Satisfied with the organisation? |
| - Information before start of project - Information stand of doctors good vs. poor | Information | - Are the GPs/HCNs/Pts well informed? - Are the MPAs informed? |
| - Reasonable effort | Expenditures | - What is your expenditure? |
| - Helpful documents | Project documents | - Were the project documents helpful? |
| - PT network meetings very positive - Continue network meetings (among participating PTs) | PT Network meetings | - Are the PT network meetings helpful? |
| - Have sufficient time for the senior | PT Time | - Satisfaction for the PT? |

| 1. Strengths and Benefits of the FPP | | |
| --- | --- | --- |
| *Meaning unit* | *Condensed Meaning Unit* | *Preliminary Questions derived from meaning units* |
| Subcategory: General and specific benefits perceived by seniors | | |
| - Feedback from the PT were very satisfactory/ useful to seniors - PT visit helpful - Seniors get different ideas - Seniors are grateful for the PT visit - We (PTs) can talk with them (the seniors) | General Benefits | - Direct benefit of seniors? - Personal visit of PT at home helpful? |
| - Detect risks - Insight in relevance of fall-prevention increased | Risk detection | - Do seniors recognize their risk of falling? - Based on the consultation by the PT? - Based on the assessments performed? |
| Subcategory: Interests of seniors | | |
| - GP or HCN recommended it - Because I trust the home care nurses - To help can be a motivation to participate - Feedback on own risks - Not having another option/feeling urged | Reasons for participation | - What is the motivation for participation? - Are these statements on the motives to participate in the FPP generally supported? |

| 2. Strengths and Benefits of the FPP *(continued)* | | |
| --- | --- | --- |
| *Meaning unit* | *Condensed Meaning Unit* | *Preliminary Questions derived from meaning units* |
| Subcategory: Further offers desired by seniors | | |
| - One month was too short for continuation of support - Longer support desired as further development of the project - Hope that the FPP will be available in the future | Continuation | - Should the support of participating seniors be continued? How? - Should the seniors be further supervised? How? - Will follow-up measures be initiated? |
| - Addresses from PTs, craftsmen or group therapies would be helpful - I would prefer to do the exercises in a group | Additional Offers | - Do seniors desire further information or additional offers, such as group therapies? |
| Subcategory: PTs instructions followed by seniors | | |
| - The PT made in situ a few changes to minimize external risk factors | Changes at home | - Were changes made at the seniors’ home? - If yes, do seniors feel more secure? |
| - I incorporate that into my exercise programme every morning | Behavioural change | - Do seniors follow PTs instructions on physical exercises? - If yes, are the exercises perceived as helpful? - Further measures such as group therapies or physiotherapy? |
| Subcategory: Project benefits perceived by GPs and HCNs | | |
| - The issue is very current and relevant | Relevance | - Statement generally supported? |
| - Effective in fall-prevention - Raises awareness / sensitisation - Identifies sources of danger / the risk of falling / individual problems of seniors - The project helps to increase seniors’ self-responsibility - Assessment at home realistic | Benefits of the FPP | - Are these statements on the FPP generally supported? |
| - Positive if outsiders evaluates it neutrally | Neutral evaluation | - Statement generally supported? |
| - Participation with low threshold and uncomplicated | Low threshold | - Statement generally supported? |
| - Feedback from the PT were very satisfactory/ useful to GPs and HCNs | Direct benefit for HCNs and GPs | - Were the feedbacks satisfactory/of use? - Any other direct benefits for HCNs and GPs? |
| - Long-term contribution important - Perseverance required | Long-term contribution | - Would GPs and HCNS support long-term continuation? |

| 1. Barriers to the inclusion of seniors (perceived by GPs and HCNs) | | |
| --- | --- | --- |
| *Meaning unit* | *Condensed Meaning Unit* | *Preliminary Questions derived from meaning units* |
| Subcategory: Lack of clarity regarding the aim of the project | | |
| - Aim of project: to prevent first falls or further falls | Aim of the project | - What is the primary aim of the project? |
| - Not important for everybody - Relevant factors: participants age, frailty, potential to benefit | Target group of seniors | - Who should be recruited? - Is the project useful for all? |
| - People were referred when they were too frail to be able to do something - Frustration of PT with regard to the timing of prevention / potential to do effective prevention - Low threshold of the exclusion criteria - Participant (frailty) dependent on the recruiter - Dementia complicates inclusion, often in combination with risk of fall | Difficulties | - How can we reach the pre-frails? / When is the correct time/stage for recruitment? - Could changes (behaviour/adaptations) be made / can progress be seen? - Depends on who is recruiting? - Satisfied that even seniors with high risk can participate? - Problem of dementia? |
| Subcategory: Procedural approach of GPs and HCNs | | |
| - Sometimes not clear how project is operated - Detailed instructions on how to recruit, standardisation of the catalogue criteria would be helpful | Understanding of project operations | - How well are the GPs informed? - Were the project operations, registration forms clear? |
| - Remembering to include participants is the issue, flyers as memo aid | Reminders | - Were reminders used to assess patients? |
| - Registered independently; self-responsibility of seniors | Responsibility for registration process | - How did seniors register? |
| - Didn’t use inclusion criteria but own observation: “Those who I had the feeling needed it” - Pre-selection by doctors makes sense, selecting suitable participants brings efficiency and saves costs | Inclusion criteria | - Is it clear how to recruit? - Is a checklist used? - Were those clearly not at risk of fall also included? - Is every patient over the age of 65 assessed and asked to participate? - Did a pre-selection take place? Which? - Efficient? / Cost saving? |
| - Many more GPs must participate / recruit | Lack of recruiting general practitioners | - Level of awareness of project? - Reasons for not registering? |

| 3. Barriers to the inclusion of seniors *(continued)* | | |
| --- | --- | --- |
| *Meaning unit* | *Condensed Meaning Unit* | *Preliminary Questions derived from meaning units* |
| Subcategory: Reasons of GPs for not recruiting seniors | | |
| - Too many different projects / lack of time - Not clear how project is operated - No registration forms were available - No perceived need / refusal by senior | Reasons for not recruiting seniors | - What were the reasons of GPs for not registering more seniors? |
| - Topic is not perceived by the public as important. - The project helps to generate public awareness of topic - Social taboo | Public relations | - Should there be more publicity on the subject of fall prevention? - Does it need more public relations work? |
| - Many offers are not near at hand in the rural area, changes and improvement are more difficult | Rural vs. town | - Is the rural environment disadvantaged compared to the town with regard to further measures? |

| 1. Barriers to participation of seniors (perceived by GPs and seniors) | | |
| --- | --- | --- |
| *Meaning unit* | *Condensed Meaning Unit* | *Preliminary Questions derived from meaning units* |
| Subcategory: Personal barriers for seniors | | |
| - Requirement to let an unknown person into the home - Lack of understanding / no perceived need - Uncertainty about new things - Another new commitment - Seniors don’t want to change anything - Costs: As long as it doesn’t cost anything seniors are happy to participate / Motivation easy because the FPP is free / Concerns about financing in the future - Some seniors no longer want to participate because they felt poorly informed - Difficulties found when no social environment exists | Potential barriers | - Why do seniors not participate? - Reasons reported by seniors or anticipated by recruiters? - Difficult for senior to let someone unknown into the home? - Would seniors have participated if it had cost something? - Were seniors well informed about the FPP? - Who made the seniors aware of the FPP? / Social support to participate in the FPP? |

| 4. Barriers to participation of seniors *(continued)* | | |
| --- | --- | --- |
| *Meaning unit* | *Condensed Meaning Unit* | *Preliminary Questions derived from meaning units* |
| Subcategory: Barriers for PTs to do assessments and give instructions | | |
| - Refusal to make changes - Seniors are sometimes a little lethargic - Contradiction: The body doesn’t want to, but I should do exercises - “Some seniors expect wonders from us” | Barriers for carrying out the FPP | - Did seniors accept changes? - Has age anything to do with inflexibility towards something new? - Will seniors continue the FPP? - Has something changed? - Could you have benefited more when you were younger? - Does it make sense to carry on with the project when mainly very frail people are recruited?--> who will be included? - What are the expectations for the project (by GPs/HCNs)? |

| 1. Barriers in interdisciplinary cooperation | | |
| --- | --- | --- |
| *Meaning unit* | *Condensed Meaning Unit* | *Preliminary Questions derived from meaning units* |
| Subcategory: Satisfaction with the SLR and with physiotherapists | | |
| - Support Swiss League Against Rheumatism (SLAR) was good - Ready to answer questions | Support | - Satisfied with support by SLAR? - Workload of SLAR? |
| - Written reports | Reports | - Were GPs satisfied with the reports? - How did S proceed with the reports? |
| Subcategory: Impact of physiotherapists work on own professional activity | | |
| - Impact of PTs recommendations | PTs recommendations | - Were PTs recommendations implemented/followed? - Were recommended further measures implemented (e.g. group therapies, further physiotherapy)? |
| Subcategory: Information and processes | | |
| - “GPs were not aware of this FPP, they somehow did not know about it.” | Information | - How well informed are GPs? - Are GPs aware of the project? - Their practice assistants? |
| - Recommendations as to how to implement in the HCN-branch were missing - How to proceed with registration forms? Many doctors give no e-mail address, they do not want e-mails | Processes | - How was the implementation in different HCN-branches and their organisation? - Do GPs/HCNs/PTs like communication by e-mail? How successful are processes? - HCNs: is interdisciplinary cooperation with GP good? - GPs: Do you receive registration forms from HCNs for signature and forwarding to SLAR and do you do so? |

| 5. Barriers in interdisciplinary cooperation *(continued)* | | |
| --- | --- | --- |
| *Meaning unit* | *Condensed Meaning Unit* | *Preliminary Questions derived from meaning units* |
| Subcategory: Satisfaction with multidisciplinarity | | |
| - Is a point of contact for GPs and HCNs - Multidisciplinary is positive because all experts are in the field - Interesting to observe how the PT does the assessments - GP cooperates with HCN and doesn’t inhibit to include senior | Multidisciplinary positive | Are these statements generally supported? |
| - Role satisfactory - HCNs: We have been working for some time with a fall protocol and have introduced measures - PT doesn’t just come, only when they are called - Areas of competences kept - Doctor as key person in the project assured - Those who were already under nursing care were declared as good | Role | - Were GPs/HCNs satisfied with their role? - Further benefits of the project? - Areas of competences kept? - Key position of GP assured? |
| - Network of trained PTs - Inclusion of clinics - Other fall prevention projects: All jump on the same topic but no cooperation | Network | - Is there an interest for addresses of trained PTs? - Should other opinion leaders (medical doctor specialists) be involved?   Question to RLS:   - Was cooperation with other organisations considered? |

For the development of the questionnaires, the categories were established under which the questions were subsequently listed.

For the participants:

1. General questions
2. General questions about the project
3. Questions about their participation
4. Questions about benefits
5. Questions about further measures
6. Questions about the right point of time for participating in the project

For the home-carers and the general practitioners:

1. General questions about the project
2. Project aims
3. Project organisation
4. Effort
5. Strengths and benefits of the project
6. Follow-up measures
7. Patient inclusion in the project
8. Interdisciplinary nature
