# Supplementary material for: Experiences of general practitioners, home care nurses, physiotherapists and seniors involved in a multidisciplinary home-based fall prevention programme: a mixed method study
Source: BMC Health Serv Res. 2016 Sep 5;16(1):469. doi: 10.1186/s12913-016-1719-5 (PMC5011799; doi:10.1186/s12913-016-1719-5)
Supplement: Additional file 2: — Tables S3-S7. All detailed questions on the topics with survey results, in addition to the Tables 3, 4, 5, 6 and 7 included in the main file. (DOCX 26 kb) [file 12913_2016_1719_MOESM2_ESM.docx]

**Tables 3 – 7: Supplementary Material**

Table 3: Detailed questions on the topic "Satisfaction with the organization and processes of the FFP" with survey results

|  | Ratings from the survey | | |
| --- | --- | --- | --- |
|  | Seniors (n = 17) “yes” n (%) | GPs (n = 25) “yes” n (%) | HCNs (n = 12) “yes” n (%) |
| Were you satisfied with the organization of the project? | 15 (88 %) | 16 (64 %) | 12 (100 %) |
| Were you well informed before the start of the project? | 15 (88 %) | 16 (64 %) | 12 (100 %) |
| Was the expenditure of time for project participation adequate? | 15 (88 %) | 25 (100 %) | 9 (75 %) |
| Was the expenditure of time for project participation an inhibition to participate longer in the project? | NA | 5 (20 %) | 4 (33 %) |
| Were the received documents helpful? | NA | 16 (64 %) | 11 (92 %) |
| Were you well informed about project operations? | NA | 14 (56 %) | 10 (83 %) |

Table 4: Subcategories (bold) of and detailed questions on the topic "Strength and benefits of the FFP" with survey results

|  | Ratings from the survey | | |
| --- | --- | --- | --- |
|  | Seniors (n = 17) “yes” n (%) | GPs (n = 25) “yes” n (%) | HCNs (n = 12) “yes” n (%) |
| **General and specific benefits perceived by seniors** |  |  |  |
| Did you attain a direct benefit out of the consultation by the PT? | 14 (82 %) | NA | NA |
| Was the personal visit of the PT at your home helpful? | 13 (76 %) | NA | NA |
| Did you recognize your own risk of falling due to the consultation by the PT? | 14 (82 %) | NA | NA |
| *If assessment had been possible:* Did you recognize the development of your danger of falling based on the assessments? | 10 (100 %) | NA | NA |
| Was your insight concerning the relevance of fall-prevention for yourself increased after participation? | 12 (71 %) | NA | NA |
| **Interests of seniors** |  |  |  |
| Why did you participate? |  |  |  |
| - *GP or HCN recommended it to you.* | 10 (59 %) | NA | NA |
| - *You recognized the danger of falling and have been motivated to do something against it actively* | 8 (47 %) | NA | NA |
| - *You wished to support the project* | 3 (18 %) | NA | NA |
| - *You have been interested in the degree of your own risk of falling* | 8 (47 %) | NA | NA |
| - *You had the assumption to not have another option* | 3 (18 %) | NA | NA |
| - *Because of your confidence in the HCN.* | 6 (35 %) | NA | NA |

Table 4: continued

|  | Ratings from the survey | | |
| --- | --- | --- | --- |
|  | Seniors (n = 17) “yes” n (%) | GPs (n = 25) “yes” n (%) | HCNs (n = 12) “yes” n (%) |
| **Further offers desired by seniors** |  |  |  |
| Do you desire a further visit by the PT at your home? | 3 (18 %) | NA | NA |
| Do you desire a regular support by a PT? | 4 (24 %) | NA | NA |
| Do you desire addresses from physiotherapists, craftsmen or group therapies? | 4 (24 %) | NA | NA |
| Would you participate again if you had the possibility to do so? | 9 (53 %) | NA | NA |
| **PTs instructions followed by seniors** |  |  |  |
| Did you investigate changes in your home after the consultation by the PT (i.e. fixating carpets or signalize door sills)? | 10 (59 %) | NA | NA |
| *If yes:* do you feel more secure in your home due to the investigated changes? | 9 (90 %) | NA | NA |
| Do you execute the instructed physical exercises received from the PT? | 10 (59 %) | NA | NA |
| Are the exercises helpful to increase physical skills (i.e. walking stairs, stand-up from chair or bed)? | 11 (65 %) | NA | NA |
| Do you carry out further measures such as group therapies or physiotherapy after the consultation by the PT? | 6 (35 %) | NA | NA |

Table 4: continued

|  | Ratings from the survey | | |
| --- | --- | --- | --- |
|  | Seniors (n = 17) “yes” n (%) | GPs (n = 25) “yes” n (%) | HCNs (n = 12) “yes” n (%) |
| **Project benefits perceived by GPs and HCNs** |  |  |  |
| Is fall-prevention in seniors more than 65 years relevant? | NA | 25 (100 %) | 12 (100 %) |
| Was the project useful to: |  |  |  |
| - *Prevent falls?* | NA | 20 (80 %) | 10 (83 %) |
| - *Draw attention to the risk of falling?* | NA | 21 (84 %) | 10 (83 %) |
| - *Detect sources of danger of falling?* | NA | 18 (72 %) | 8 (67 %) |
| - *Identify the risk of falling?* | NA | 10 (40 %) | 8 (67 %) |
| - *Identify the individual problems of seniors?* | NA | 8 (32 %) | 7 (58 %) |
| - *Increase personal responsibility of seniors?* | NA | 7 (28 %) | 7 (58 %) |
| Was the neutral evaluation by the PT positive? | NA | 10 (40 %) | 10 (83 %) |
| Did the project have a low threshold and was it uncomplicated? | NA | 12 (48 %) | 6 (50 %) |
| Did you draw a direct benefit for yourself from the project? | NA | 7 (28 %) | 4 (33 %) |
| Would you support a long-term continuation of the project? | NA | 15 (60 %) | 10 (83 %) |

Table 5: Subcategories (bold) of and detailed questions on the topic "Barriers to the inclusion of seniors" with survey results

|  | Ratings from the survey | | |
| --- | --- | --- | --- |
|  | Seniors (n = 17) “yes” n (%) | GPs (n = 25) “yes” n (%) | HCNs (n = 12) “yes” n (%) |
| **Lack of clarity regarding the aim of the project** |  |  |  |
| What is the primary aim of the project (one answer): |  |  |  |
| - *The prevention of first falls.* | NA | 20 (80 %) | 4 (33 %) |
| - *The prevention of further falls.* |  | 6 (24 %) | 8 (67 %) |
| Did you encourage seniors without previous interventions in fall prevention? | NA | 16 (64 %) | 10 (83 %) |
| Did you encourage seniors with previous interventions in fall prevention? | NA | 9 (35 %) | 8 (67 %) |
| Would you have profited more by the project if you were younger? | 7 (41 %) | NA | NA |
| Would you have participated if you were younger? | 7 (41 %) | NA | NA |
| **Procedural approach of GPs and HCNs** |  |  |  |
| Did you recruit at least one senior? | NA | 12 (48 %) | 12 (100 %) |
| Did you know how to recruit seniors? | NA | 21 (84 %) | 12 (100 %) |
| Did you use reminders (i.e. flyer, post-it…)? | NA | 4 (16 %) | 4 (33 %) |
| Did your patients sign up for the project by themselves? | NA | 5 (42 %) | NA |
| Did you choose following selection criteria (multiple answers possible)? | NA |  |  |
| - *Age > 70* | NA | 3 (12 %) | 1 (8 %) |
| - *Known falls* | NA | 11 (44 %) | 8 (67 %) |
| - *Obvious risk of falling* | NA | 19 (76 %) | 8 (67 %) |
| - *Mobility problems of seniors* | NA | 14 (56 %) | 9 (75 %) |

Table 5: Continued

|  | Ratings from the survey | | |
| --- | --- | --- | --- |
|  | Seniors (n = 17) “yes” n (%) | GPs (n = 25) “yes” n (%) | HCNs (n = 12) “yes” n (%) |
| **Reasons of GPs for not recruiting seniors** |  |  |  |
| Why did you not recruit any seniors? (n = 13) |  |  |  |
| - *Expenditure of time for project participation* | NA | 1 (8 %) | NA |
| - *Project operations were not clear* | NA | 4 (31 %) | NA |
| - *No registration forms were available* | NA | 4 (31 %) | NA |
| - *No perceived need / refusal by senior* | NA | 10 (77 %) | NA |
| Is the increase of public relations on the topic fall-prevention important? | NA | 87 % | 7 (58 %) |

Table 6: Subcategories (bold) of and detailed questions on the topic "Barriers to participation" with survey results

|  | Ratings from the survey | | |
| --- | --- | --- | --- |
|  | Seniors (n = 17) “yes” n (%) | GPs (n = 25) “yes” n (%) | HCNs (n = 12) “yes” n (%) |
| **Personal barriers for seniors** |  |  |  |
| Did you feel urged to participate? | 1 (6 %) | NA | NA |
| Did you have difficulties with being consulted by the PT at your home? | 1 (6 %) | NA | NA |
| Had you participated in the project in case you had to pay for it? | 6 (35 %) | NA | NA |
| **Barriers for PTs to do asssessments and give instructions** |  |  |  |
| Was it possible to perform physical assessments to obtain your risk of falling? | 10 (59 %) | NA | NA |

Table 7: Subcategories (bold) of and detailed questions on the topic "Barriers in interdisciplinary cooperation" with survey results

|  | Ratings from the survey | | |
| --- | --- | --- | --- |
|  | Seniors (n = 17) “yes” n (%) | GPs (n = 25) “yes” n (%) | HCNs (n = 12) “yes” n (%) |
| **Satisfaction with the SLR and with physiotherapists** |  |  |  |
| Were you satisfied with the support by the “Swiss League against Rheumatism”? | NA | 10 (40 %) | 8 (67 %) |
| Were you satisfied with the report received from PTs? | NA | 9 (36 %) | 6 (50 %) |
| **Impact of physiotherapists work on own professional activity** |  |  |  |
| Did you partially or in general implement the recommendations by the PTs? | NA | 11 (44 %) | 7 (58 %) |
| Did you initiate further measures (i.e. group therapies, further physiotherapy)? | NA | 6 (24 %) | 2 (17 %) |
| **Information and processes** |  |  |  |
| Do you think GPs were well informed? | NA | NA | 6 (50 %) |
| Did you know that HCNs were involved in the project? | NA | 23 (93 %) | NA |
| Did you receive some recommendations from HCNs to register seniors? | NA | 2 (8 %) | NA |
| Was the project feasible within your HCN-branch? | NA | NA | 11 (92 %) |
| Were your medical practice assistants informed? | NA | 8 (32 %) | NA |
| **Satisfaction with multidisciplinarity** |  |  |  |
| Was it positive that the project was multidisciplinary? | NA | 9 (36 %) | 4 (33 %) |
| Were the areas of expertise between disciplines not mixed? | NA | 4 (16 %) | 5 (42 %) |
| Is the interdisciplinary cooperation generally good? | NA | 8 (32 %) | 5 (42 %)NA |
| Were you satisfied with the role allocation in the project? | NA | 15 (60 %) | 8 (67 %) |
| Would you support the participation of e.g. rehabilitation centers or hospitals in the project? | NA | 9 (36 %) | 9 (75 %) |
